# Associations between the “Double Reduction” policy and physical activity and overweight/obesity among Chinese school-aged children: a quasi-experimental difference-in-differences analysis

**DOI:** 10.3389/fpubh.2026.1963763

**Published:** 2026-10-14

**Authors:** Wenhui Yu, Bing Zhang

**Affiliations:** Institute of Physical Education, Huanggang Normal University, Huanggang, Hubei, China

**Keywords:** child health, China Family Panel Studies, difference-in-differences, Double Reduction policy, obesity, overweight, physical activity, quasi-experimental design

## Abstract

**Background:**

In July 2021, China implemented the “Double Reduction” policy to ease homework burden and curb off-campus tutoring for students in compulsory education. The policy has been evaluated mainly for academic and mental health outcomes; whether it coincided with changes in physical activity and weight status is unclear. This study used the policy as a natural experiment to examine associations between the Double Reduction policy, moderate-to-vigorous physical activity (MVPA), and overweight/obesity among Chinese school-aged children.

**Methods:**

We analyzed the 2018, 2020, and 2022 waves of the China Family Panel Studies (CFPS) in a quasi-experimental difference-in-differences (DiD) design. At each wave, students enrolled in compulsory education (grades 1–9) formed the treatment group and senior high school students (grades 10–12), who were not covered by the policy, formed the comparison group. The analytical sample comprised 4,237 students contributing 10,799 person-wave observations. Outcomes were self-reported weekly MVPA minutes and overweight/obesity defined by age- and sex-specific body mass index z-scores (WHO 2007 reference). Event-study specifications, propensity-score weighting, longitudinal survey weights, and placebo tests assessed the stability of the estimates.

**Results:**

The policy was associated with 43.1 additional MVPA minutes per week (95% CI: 33.8–52.3; *p* < 0.001) and a 3.9 percentage-point reduction in overweight/obesity (95% CI: −7.2 to −0.6; *p* = 0.022) among compulsory education students relative to the comparison group. Estimates were larger among primary school students and among children whose parents had lower educational attainment. The 2018 pre-policy interaction was small and not statistically significant for either outcome, although two pre-policy waves offer limited power for testing pre-trends. Estimates were similar under balanced-panel, propensity-score-weighted, survey-weighted, and placebo specifications.

**Conclusion:**

The Double Reduction policy coincided with measurable improvements in physical activity and weight status among Chinese school-aged children. Education policies that relieve academic burden may carry collateral public health benefits and deserve explicit consideration in child health strategies.

## 1 Introduction

Physical inactivity and childhood obesity rank among the most pressing public health challenges of the twenty-first century (1–3). The World Health Organization (WHO) estimates that more than 80% of adolescents worldwide fail to meet the recommended 60 min of daily moderate-to-vigorous physical activity (MVPA) (1), while the global prevalence of childhood overweight and obesity has risen steadily over the past four decades (4). China mirrors these trends. The 2022 China Report Card on physical activity for children and adolescents assigned an overall grade of “D” (5), and national surveillance data indicate that roughly 20% of school-aged children are overweight or obese, with rates still climbing (6, 7).

The determinants of physical inactivity and obesity in childhood are multifactorial, spanning individual, family, school, community, and policy levels (8, 9). Among these, the school environment, including homework load, structured physical education, recess time, and extracurricular demands, plays a particularly influential role (10). Chinese students have historically faced one of the heaviest academic workloads in the world, spending an average of 3 to 5 hours daily on homework and off-campus tutoring, which substantially constrains the time available for physical activity and active play (5, 11).

In July 2021, the General Office of the Central Committee of the Communist Party of China and the General Office of the State Council jointly issued the “Opinions on Further Reducing the Burden of Homework and Off-Campus Training for Students in Compulsory Education,” commonly referred to as the “Double Reduction” (Shuang Jian) policy (12). The policy mandates strict limits on homework quantity (no written homework for grades 1–2, at most 60 min for grades 3–6, and at most 90 min for grades 7–9), prohibits for-profit academic tutoring during weekends and holidays, and requires schools to provide after-school services that include physical activity and arts programs. By the end of 2021, more than 90% of off-campus subject-based tutoring institutions had closed or converted to non-profit status (13).

The Double Reduction policy offers a rare natural experiment for studying how a substantial reduction in academic burden relates to children’s health behaviors, and early evaluations have begun to appear. Liu and Wang (13) documented increases in sports participation and curriculum resource utilization in primary schools after implementation. Wang et al. (14) reported reductions in depressive and anxiety symptoms among adolescents, and Zhou and Fan (15) found improvements in subjective well-being among primary school students. To date, however, no study has used nationally representative longitudinal data and a quasi-experimental design to examine whether the policy coincided with changes in physical activity levels and overweight/obesity prevalence.

The present study addresses this gap by applying a difference-in-differences (DiD) framework to the China Family Panel Studies (CFPS). We treat the Double Reduction policy as an exogenous shock that differentially affected students enrolled in compulsory education (grades 1–9) and senior high school students (grades 10–12), who were not subject to the policy. Using panel waves from the pre-policy (2018, 2020) and post-policy (2022) periods, we estimate the policy’s association with two outcomes: weekly MVPA minutes and overweight/obesity status. We hypothesize that the policy is associated with higher physical activity and lower overweight/obesity among compulsory education students relative to the comparison group, and we explore whether these associations vary by education stage, sex, and parental education.

## 2 Methods

### 2.1 Study design

This study employs a quasi-experimental difference-in-differences design to estimate the association between the Double Reduction policy and the two health outcomes (16, 17). The DiD approach compares changes in outcomes over time between students affected by the policy and students not affected, netting out time-invariant unobserved differences between groups and common secular trends. The identifying assumption is that, in the absence of the policy, the two groups would have followed parallel outcome trends.

Treatment status is defined by the grade in which a student was enrolled at each survey wave, because the policy applies to compulsory education (grades 1–9) and exempts senior high school (grades 10–12). Grade enrollment changes as children age, so treatment status is time-varying: a student in grade 8 in 2018 was exposed to the policy in 2022 only if still enrolled in a covered grade. In our sample, 867 students who were in compulsory education in 2018 had progressed to senior high school by 2022 and therefore moved from treatment to comparison status, while no student moved in the opposite direction. The estimates are therefore intention-to-treat effects of being enrolled in a policy-covered grade in the post-policy period. The pre-policy period comprises the 2018 and 2020 waves and the post-policy period the 2022 wave; the two pre-policy waves permit an event-study specification and a formal, if low-powered, check on differential pre-trends.

### 2.2 Data source and study sample

Data were drawn from the CFPS, a nationally representative longitudinal survey administered by the Institute of Social Science Survey at Peking University (18). The CFPS uses multistage probability-proportional-to-size sampling with implicit stratification, covering 25 provinces, municipalities, and autonomous regions and approximately 95% of the Chinese population, and collects information at the individual, household, and community levels.

We used the 2018, 2020, and 2022 waves. Figure 1 summarizes the sample flow. The initial sample comprised 6,842 children and adolescents aged 6–18 years with at least one wave of observation. We excluded 1,762 participants with missing data on key variables (physical activity, height or weight, grade enrollment, or covariates) and 843 participants observed in only one wave, because individual fixed-effects estimation requires at least two observations per person. The analytical sample consisted of 4,237 students contributing 10,799 person-wave observations (3,937 in 2018, 4,237 in 2020, and 2,625 in 2022). A balanced-panel subsample of 2,325 students observed in all three waves (6,975 observations) was used in sensitivity analyses.

**Figure 1:**
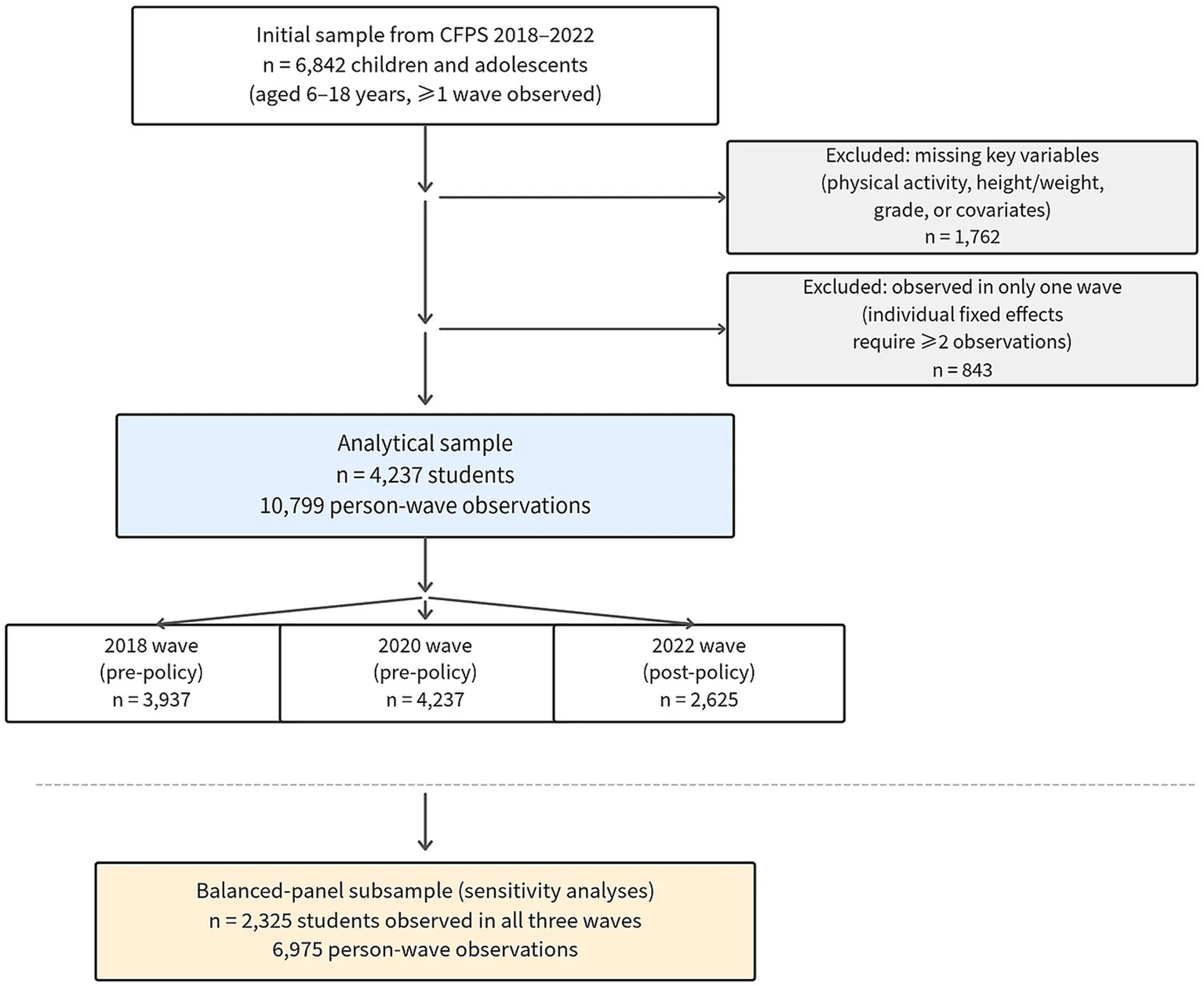
Flow of participants into the analytical sample. CFPS, China Family Panel Studies.

### 2.3 Measures

#### 2.3.1 Physical activity

The CFPS exercise items differ across waves, and we harmonized them to a common weekly metric. In the 2018 wave, respondents, or caregivers of children younger than 10 years, reported the total duration of physical exercise in the past week, recorded in hours and converted to minutes. In the 2020 and 2022 waves, the survey asked how often the respondent had taken part in physical exercise in the past month (item QP701N; eight categories) and the usual duration of each exercise session (item QP702N; minutes). We assigned weekly session frequencies to the categories (never = 0; less than once a month = 0.5; about once a month = 1.5; one to two times a week = 1.5; three to four times a week = 3.5; five or more times a week = 5.5; once a day = 7; twice a day or more = 14) and multiplied frequency by session duration to obtain weekly exercise minutes. Following the WHO and the Chinese expert consensus on child physical activity, MVPA refers to activities that markedly raise heart rate and breathing, such as running, ball games, swimming, and brisk cycling; walking counted only when reported as exercise (1, 11). Values above the 99th percentile (980 min/week) were winsorized. Because the 2018 item used a past-week recall and the later waves a past-month frequency format, the harmonized weekly totals are not perfectly comparable across waves; we return to this point in the limitations.

#### 2.3.2 Overweight and obesity

Height and weight were reported by respondents or, for younger children, by their primary caregivers. Body mass index (BMI) was converted to age- and sex-specific z-scores using the WHO 2007 growth reference for children and adolescents aged 5–19 years, and students with a BMI z-score above +1 SD were classified as overweight or obese, consistent with recent Chinese surveillance studies (8, 19).

#### 2.3.3 Treatment and post-policy indicators

The treatment indicator equals 1 for students enrolled in grades 1–9 at the survey wave and 0 for students enrolled in grades 10–12. The post-policy indicator equals 1 for the 2022 wave. The coefficient of interest is the interaction between the two.

#### 2.3.4 Covariates

Time-varying covariates comprised age (years), hukou status (urban vs. rural), log-transformed annual household income per capita, parental education (highest level attained by either parent, dichotomized at junior secondary or below vs. senior secondary or above), and school type (public vs. private). Sex was time-invariant. Because age increases mechanically with the survey wave, age and wave indicators are collinear once individual fixed effects are included; we therefore did not enter age as a separate covariate in the main models and instead probed age-related confounding with flexible age-by-wave interactions in a dedicated specification (see below).

### 2.4 Statistical analysis

Descriptive statistics were computed for each wave, stratified by treatment status. The primary model was a two-way fixed-effects specification, Yit = *α* + *β*(Treatit × Postt) + γ′Xit + μi + λt + εit, where Yit is the outcome for student i in wave t, μi denotes individual fixed effects, λt denotes wave fixed effects, Xit is the vector of time-varying covariates, and *β* is the coefficient of interest. Standard errors were clustered at the individual level (16). Weekly MVPA minutes were modeled with linear fixed-effects regression, and overweight/obesity with a linear probability model, which facilitates the interpretation of coefficients as percentage-point changes and accommodates individual fixed effects.

To examine pre-policy trends, we estimated an event-study specification that interacted the treatment indicator with wave dummies, using 2020 as the reference wave (17). A small and statistically non-significant treatment-by-2018 interaction is consistent with parallel pre-policy trends, although two pre-policy waves provide limited power to detect differential trends.

Several sensitivity analyses probed the stability of the estimates. First, we re-estimated the models on the balanced-panel subsample. Second, we excluded the 2020 wave (collected during the COVID-19 outbreak) and, separately, the 2018 wave. Third, we conducted a placebo test that assigned a false policy date of 2020 and compared 2018 with 2020, both genuinely pre-policy waves. Fourth, we combined DiD with propensity-score weighting. Because grade enrollment, and therefore age, defines treatment assignment, matching on age would discard the identifying variation; we instead estimated the propensity score at baseline from sex, hukou status, log household income, parental education, and school type, and applied inverse-probability weights trimmed at the 99th percentile. Balance was assessed with standardized mean differences. Fifth, we applied CFPS longitudinal survey weights. Sixth, to probe confounding by age-specific outcome trajectories, we added interactions between a continuous age term and the wave indicators. Finally, we estimated subgroup models by education stage, sex, and parental education, and tested group differences formally with triple-interaction terms. Analyses were performed in Stata 17.0 (StataCorp LLC, College Station, TX, USA), with two-sided *α* = 0.05.

## 3 Results

### 3.1 Sample characteristics

Table 1 reports wave-specific descriptive statistics. At baseline in 2018, students in the treatment group were younger than comparison students (mean age 11.7 vs. 16.0 years), a mechanical consequence of defining treatment by grade enrollment. The groups were otherwise similar: the shares of boys (51.3% vs. 51.5%), urban hukou (44.7% vs. 45.2%), log household income (9.55 vs. 9.51), low parental education (63.6% vs. 65.1%), and private school enrollment (6.2% vs. 6.6%) differed little. Baseline weekly MVPA averaged 186.9 min in the treatment group and 175.3 min in the comparison group, and overweight/obesity prevalence was 21.1 and 23.1%, respectively. Between 2018 and 2022, mean MVPA rose from 186.9 to 233.9 min/week in the treatment group and from 175.3 to 188.3 min/week in the comparison group, while overweight/obesity prevalence moved from 21.1 to 20.9% and from 23.1 to 24.1%, respectively.

**Table 1:** Sample characteristics by survey wave and treatment status.

Characteristic	2018 (pre-policy)		2020 (pre-policy)		2022 (post-policy)	
	Treated	Comparison	Treated	Comparison	Treated	Comparison
*n*	3,131	806	2,335	1,902	1,758	867
Age, years, mean (SD)	11.7 (2.8)	16.0 (0.5)	11.6 (2.2)	17.3 (0.9)	12.6 (1.7)	17.0 (1.0)
Male, %	51.3	51.5	51.9	50.3	50.6	54.0
Urban hukou, %	44.7	45.2	45.4	44.3	44.4	46.5
Log household income per capita, mean (SD)	9.55 (0.50)	9.51 (0.51)	9.55 (0.50)	9.52 (0.50)	9.55 (0.50)	9.55 (0.49)
Low parental education, %	63.6	65.1	64.8	63.8	65.4	61.9
Private school, %	6.2	6.6	6.4	6.3	6.9	6.1
MVPA, min/week, mean (SD)	186.9 (109.9)	175.3 (106.3)	193.5 (106.3)	184.7 (107.9)	233.9 (109.6)	188.3 (108.0)
Overweight/obesity, %	21.1	23.1	21.7	22.2	20.9	24.1

Treated students were enrolled in compulsory education (grades 1–9) at the survey wave; comparison students were enrolled in senior high school (grades 10–12). Group sizes change across waves because treatment status is defined by current grade and students progress through grades; 867 students moved from treatment in 2018 to comparison status in 2022. MVPA, moderate-to-vigorous physical activity; SD, standard deviation. Low parental education denotes junior secondary schooling or below for the more educated parent.

### 3.2 Main difference-in-differences estimates

Table 2 presents the main results. After adjustment for individual and wave fixed effects and time-varying covariates, enrollment in a policy-covered grade after the Double Reduction policy was associated with 43.1 additional MVPA minutes per week (95% CI: 33.8–52.3; *p* < 0.001), equivalent to about 23% of the pre-policy mean in the treatment group. Overweight/obesity prevalence fell by 3.9 percentage points (95% CI: −7.2 to −0.6; *p* = 0.022), a relative reduction of about 18% from the baseline prevalence of 21.1%.

**Table 2:** Difference-in-differences estimates of the association between the Double Reduction policy and study outcomes, main and sensitivity specifications.

Specification	MVPA, min/week, *β* (95% CI)	*p*	Overweight/obesity, *β* (95% CI)	*p*
Main two-way fixed effects	43.1 (33.8 to 52.3)	< 0.001	−0.039 (−0.072 to −0.006)	0.022
Balanced panel (*n* = 2,325)	42.8 (33.3 to 52.3)	< 0.001	−0.034 (−0.068 to 0.001)	0.054
Excluding the 2020 wave	42.3 (31.3 to 53.3)	< 0.001	−0.039 (−0.078 to 0.001)	0.057
Excluding the 2018 wave	43.6 (33.2 to 54.1)	< 0.001	−0.036 (−0.074 to 0.002)	0.061
Placebo: false policy year 2020	0.1 (−7.9 to 8.1)	0.982	−0.013 (−0.042 to 0.017)	0.400
Propensity-score weighted	42.8 (33.3 to 52.3)	< 0.001	−0.034 (−0.068 to 0.001)	0.054
CFPS longitudinal weights	42.0 (32.1 to 52.0)	< 0.001	−0.039 (−0.075 to −0.004)	0.031
Flexible age-by-wave trends	26.9 (10.6 to 43.1)	0.001	−0.033 (−0.092 to 0.026)	0.279
Event study: treat × 2022	42.9 (33.6 to 52.1)	< 0.001	−0.039 (−0.073 to −0.006)	0.022

All specifications include individual and wave fixed effects and time-varying covariates (hukou status, log household income, parental education, school type); standard errors are clustered at the individual level. The analytical sample comprises 4,237 students and 10,799 person-wave observations unless stated otherwise. The placebo row assigns a false policy date of 2020 and compares the 2018 and 2020 waves. Propensity-score weighting uses baseline sex, hukou status, log household income, parental education, and school type, with inverse-probability weights trimmed at the 99th percentile; all post-weighting standardized mean differences were below 0.01. The event-study row uses 2020 as the reference wave; the treat × 2018 coefficient was −0.8 (*p* = 0.713) for MVPA and −0.002 (*p* = 0.846) for overweight/obesity. CI, confidence interval; MVPA, moderate-to-vigorous physical activity.

### 3.3 Event study and sensitivity analyses

The event-study estimates are plotted in Figure 2. The treatment-by-2018 interaction, measured against the 2020 reference wave, was small and not statistically significant for MVPA (*β* = −0.8; 95% CI: −5.3 to 3.6; *p* = 0.713) or overweight/obesity (*β* = −0.002; 95% CI: −0.018 to 0.015; *p* = 0.846), and the treatment-by-2022 interactions were significant for both outcomes (MVPA: *β* = 42.9; 95% CI: 33.6–52.1; *p* < 0.001; overweight/obesity: *β* = −0.039; 95% CI: −0.073 to −0.006; *p* = 0.022). These patterns are consistent with parallel pre-policy trends, but with only two pre-policy waves the test has limited power, so we interpret it as supportive rather than definitive.

**Figure 2:**
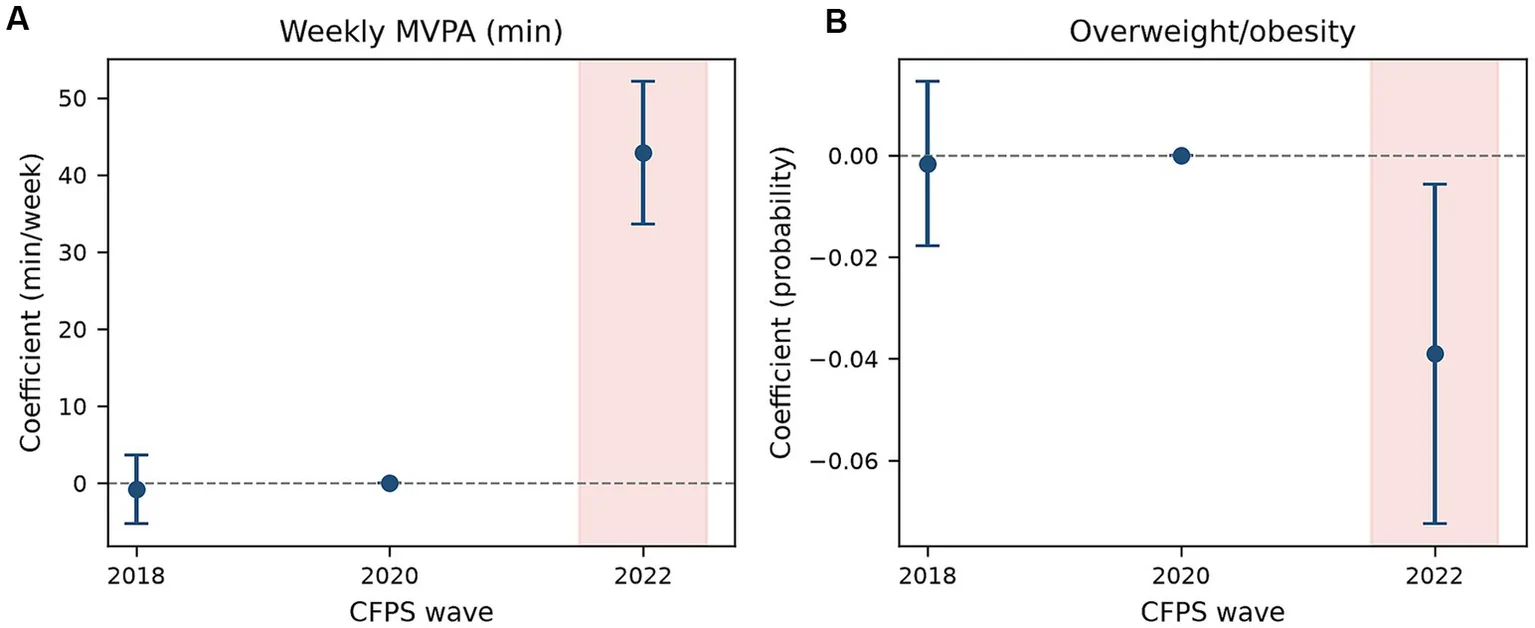
Event-study estimates of the association between the Double Reduction policy and **(A)** weekly minutes of moderate-to-vigorous physical activity and **(B)** overweight/obesity. Points show the coefficients on interactions between the treatment indicator and wave dummies with 2020 as the reference wave; whiskers show 95% confidence intervals from individual-clustered standard errors. Models include individual and wave fixed effects and time-varying covariates. The shaded region marks the post-policy period.

Sensitivity analyses are summarized in Table 2. The balanced-panel subsample yielded nearly identical MVPA estimates (*β* = 42.8; 95% CI: 33.3–52.3; *p* < 0.001). Excluding either the 2020 or the 2018 wave left the MVPA estimate essentially unchanged (42.3 and 43.6 min/week, respectively). The placebo test, which pretended the policy had taken effect in 2020, returned estimates close to zero for both outcomes (MVPA: *β* = 0.1; 95% CI: −7.9 to 8.1; *p* = 0.982; overweight/obesity: *β* = −0.013; 95% CI: −0.042 to 0.017; *p* = 0.400), indicating that the main estimates are specific to the actual policy window. After propensity-score weighting, all standardized mean differences for the matching covariates were below 0.01, and the weighted DiD estimate for MVPA was 42.8 min/week (95% CI: 33.3–52.3; *p* < 0.001). Applying CFPS longitudinal weights gave 42.0 min/week (95% CI: 32.1–52.0; *p* < 0.001). The overweight/obesity estimates remained negative and close in magnitude across these specifications (−3.4 to −3.9 percentage points), with confidence intervals that crossed zero in some of them (p between 0.031 and 0.061). Adding flexible age-by-wave interactions attenuated the MVPA estimate to 26.9 min/week (95% CI: 10.6–43.1; *p* = 0.001) and the overweight/obesity estimate to −0.033 (95% CI: −0.092 to 0.026; *p* = 0.279); we discuss this specification in the limitations.

### 3.4 Subgroup analyses

Table 3 reports subgroup estimates together with formal interaction tests. The MVPA association was larger for primary school students (*β* = 58.2; 95% CI: 45.1–71.3) than for junior secondary students (*β* = 36.8; 95% CI: 19.7–54.0), and the difference was significant (interaction *β* = 23.7; 95% CI: 12.8–34.5; *p* < 0.001). The association was also larger among students whose parents had lower educational attainment (*β* = 52.1; 95% CI: 40.6–63.5) than among students with more educated parents (*β* = 26.8; 95% CI: 11.1–42.4; interaction *β* = −19.2; 95% CI: −30.5 to −7.9; *p* < 0.001). Estimates for boys (*β* = 50.8) and girls (*β* = 34.4) did not differ significantly (interaction *p* = 0.307). For overweight/obesity, the reduction was concentrated among boys (*β* = −0.069; 95% CI: −0.117 to −0.022; *p* = 0.004), whereas the estimate for girls was close to zero (*β* = −0.004; *p* = 0.852); the formal interaction was not significant (*p* = 0.195), so this sex difference should be read as suggestive. Differences by education stage and parental education in the overweight/obesity outcome were not significant (interaction *p* = 0.276 and *p* = 0.128, respectively).

**Table 3:** Subgroup difference-in-differences estimates with formal interaction tests.

Subgroup	MVPA, min/week, *β* (95% CI)	*p*	Overweight/obesity, *β* (95% CI)	*p*
Primary school (grades 1–6)	58.2 (45.1 to 71.3)	< 0.001	−0.040 (−0.090 to 0.009)	0.111
Junior secondary (grades 7–9)	36.8 (19.7 to 54.0)	< 0.001	−0.019 (−0.079 to 0.042)	0.545
Primary vs. junior interaction	23.7 (12.8 to 34.5)	< 0.001	−0.021 (−0.058 to 0.016)	0.276
Boys	50.8 (38.1 to 63.5)	< 0.001	−0.069 (−0.117 to −0.022)	0.004
Girls	34.4 (20.8 to 48.0)	< 0.001	−0.004 (−0.051 to 0.042)	0.852
Girls vs. boys interaction	−5.7 (−16.6 to 5.2)	0.307	0.024 (−0.013 to 0.061)	0.195
Low parental education	52.1 (40.6 to 63.5)	< 0.001	−0.029 (−0.071 to 0.012)	0.169
High parental education	26.8 (11.1 to 42.4)	< 0.001	−0.058 (−0.115 to −0.002)	0.044
High vs. low education interaction	−19.2 (−30.5 to −7.9)	< 0.001	−0.031 (−0.071 to 0.009)	0.128

Each subgroup row reports the treat × post coefficient from a two-way fixed-effects model estimated within the subgroup. Interaction rows report the triple-interaction coefficient from pooled models (treat × post × subgroup indicator); for sex and education stage the interaction contrasts the second group with the first. Low parental education denotes junior secondary schooling or below for the more educated parent. CI, confidence interval; MVPA, moderate-to-vigorous physical activity.

## 4 Discussion

In this quasi-experimental analysis of nationally representative panel data, the Double Reduction policy was associated with about 43 additional minutes of MVPA per week and a 3.9 percentage-point reduction in overweight/obesity among students enrolled in compulsory education. The estimates were stable across balanced-panel, propensity-score-weighted, survey-weighted, and placebo specifications, and the largest gains appeared among primary school students and children whose parents had less education.

### 4.1 Interpretation of the findings

The magnitude of the physical activity association is meaningful for public health. Children and adolescents are advised to accumulate at least 60 min of MVPA per day, roughly 420 min per week (1, 9), and the estimated increase of 43 min corresponds to about 10% of that weekly recommendation. Against a backdrop in which more than 80% of adolescents worldwide fall short of the recommendation, even a policy-induced shift of this size, sustained across a large population, could translate into appreciable health gains.

Several mechanisms could account for the observed associations, although our data cannot adjudicate among them and we offer them as hypotheses. First, the reduction in homework and the elimination of weekend tutoring freed discretionary time that children may have reallocated to active play and organized sports (13, 15). Second, the policy required schools to run after-school services, and structured programs of this kind can raise physical fitness and activity levels when delivered well (20). Third, lower academic pressure may have improved psychological well-being (14), which is linked in turn to greater engagement in physical activity (10). Fourth, time previously spent in online tutoring sessions was sedentary screen time, so its removal may have improved energy balance through a second channel.

The larger associations among primary school students are consistent with two considerations. Younger children display greater behavioral plasticity and may respond more readily when discretionary time expands (21), and the policy’s strictest provisions (no written homework in grades 1–2 and tight time caps in grades 3–6) target exactly those years. Junior secondary students remain covered by the policy, but the high school entrance examination (Zhongkao) looms over their daily schedules and may blunt the release of leisure time. The gradient by parental education points in an equity-relevant direction. Before the policy, access to organized sports in China tracked family resources, since wealthier parents could purchase private coaching and club memberships (6, 19, 22). Universally available school-based after-school programs may have narrowed that gap by giving disadvantaged children structured opportunities they previously lacked, although we cannot verify this channel directly.

### 4.2 Comparison with prior literature

Our findings extend the early evidence on the Double Reduction policy. Liu and Wang (13) described increased sports participation in primary schools with cross-sectional and qualitative data, and Wang et al. (14) and Zhou and Fan (15) documented mental health and well-being gains. The present analysis adds longitudinal, quasi-experimental evidence on physical activity and weight status, two outcomes these studies did not examine.

International evidence on school schedule disruptions offers an instructive mirror image. During COVID-19 school closures in the United Kingdom, children’s physical activity fell and body mass index rose, plausibly because structured school-time activity disappeared (23). The Double Reduction policy operated in the opposite direction: it kept schools open while expanding the space for activity within and around the school day, and we observe changes consistent with that reversal. More broadly, the results speak to the “Health in All Policies” perspective endorsed by the WHO (1): a reform designed purely for educational ends was accompanied by measurable changes in child health behaviors and outcomes.

### 4.3 Policy implications

Three implications follow. First, the health-related associations documented here strengthen the case for sustaining the Double Reduction policy and argue for including child health indicators in its formal evaluation framework, in line with calls to treat physical activity promotion as a cross-sector public health priority (24). Second, the larger gains among children of less-educated parents suggest that school-based after-school services are reaching populations that market-based provision tended to bypass; maintaining funding and monitoring for these programs would protect that equity dividend. Third, the weaker associations in junior secondary school indicate that examination pressure can crowd out activity time for older students, so targeted measures, such as protected daily activity periods, may be needed for that group. The quality of after-school physical activity provision also matters: structured curricula, trained physical education staff, and parental engagement have improved fitness outcomes in pilot programs and could amplify the policy’s health dividends (10, 20).

### 4.4 Limitations

Several limitations qualify these findings. First, physical activity was self-reported or caregiver-reported rather than device-measured, which invites recall and social desirability bias; the CFPS exercise items have nonetheless been used widely in Chinese population health research (18, 19). Second, the exercise questions changed across waves, from a past-week duration format in 2018 to a past-month frequency-by-duration format in 2020 and 2022. We harmonized the items to weekly totals, but the recall windows differ, and any resulting measurement error would most plausibly attenuate the estimates. Third, height and weight were reported rather than measured, so overweight/obesity prevalence is likely understated, although reporting error would have to differ systematically between groups and waves to generate the observed pattern.

Fourth, treatment and comparison students differ in age by construction, since grade enrollment defines treatment. We addressed this with individual fixed effects, propensity-score weighting on non-age covariates, and flexible age-by-wave interactions. The MVPA association survived the flexible age specification, but the overweight/obesity estimate was attenuated and its confidence interval included zero, so we cannot rule out age-related confounding for the weight outcome. Fifth, with only two pre-policy waves, the event-study test of parallel trends has limited power. Sixth, the 2022 wave was collected while COVID-19 control measures were still in place in parts of China, and the pandemic disrupted children’s physical activity in many settings (23, 25). Excluding the 2020 wave did not change the estimates, but the 2022 measurements themselves may partly reflect pandemic conditions, and residual confounding is possible. Seventh, the post-policy follow-up covers roughly 1 year, so we cannot say whether the associations persist. Finally, the design identifies intention-to-treat associations of grade-level policy exposure; we observe neither individual compliance nor the dosage of specific policy components, and unmeasured time-varying confounders cannot be excluded. Work that exploits grade-eligibility thresholds in a regression discontinuity design, or that collects device-measured activity prospectively, would sharpen causal inference.

## 5 Conclusion

The Double Reduction policy, an educational reform designed to relieve academic burden, was associated with about 43 additional minutes of weekly MVPA and a 3.9 percentage-point reduction in overweight/obesity among Chinese students in compulsory education. Associations were strongest for primary school students and for children whose parents had less education, hinting at an equity dividend. These results illustrate how education policy and child health are linked, and they argue for evaluating future educational reforms with health outcomes in view.

## Data Availability

Publicly available datasets were analyzed in this study. This data can be found here: repository: China Family Panel Studies (CFPS), Institute of Social Science Survey, Peking University Direct link: http://www.isss.pku.edu.cn/cfps/.
